# Emerging role of IRE1α in vascular diseases

**DOI:** 10.1002/ccs3.12056

**Published:** 2024-11-10

**Authors:** Jia Shi, Fan He, Xiaogang Du

**Affiliations:** ^1^ Department of Nephrology The First Affiliated Hospital of Chongqing Medical University Chongqing China; ^2^ Department of Nephrology Tongji Hospital Affiliated to Tongji Medical College Huazhong University of Science and Technology Wuhan Hubei Province China

**Keywords:** atherosclerosis, IRE1α, vascular disease, XBP1

## Abstract

A mounting body of evidence suggests that the endoplasmic reticulum stress and the unfolded protein response are involved in the underlying mechanisms responsible for vascular diseases. Inositol‐requiring protein 1α (IRE1α), the most ancient branch among the UPR‐related signaling pathways, can possess both serine/threonine kinase and endoribonuclease (RNase) activity and can perform physiological and pathological functions. The IRE1α‐signaling pathway plays a critical role in the pathology of various vascular diseases. In this review, we provide a general overview of the physiological function of IRE1α and its pathophysiological role in vascular diseases.

## Graphical abstract

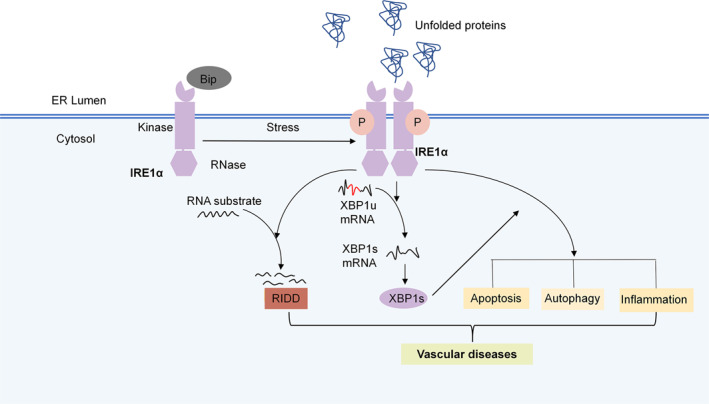

## 1 INTRODUCTION

Cardiovascular diseases (CVDs) are the leading cause of morbidity and mortality worldwide. According to the World Health Organization, 32% of all global deaths worldwide are due to CVD.^1^ Vascular diseases, including atherosclerosis, systemic and pulmonary hypertension, neointimal hyperplasia, vascular calcification, aneurysms, and other disorders, are common risk factors for overall mortality. The pathology of vascular diseases is closely related to the dysfunction of the vasculature and vascular cells. The arterial wall contains three layers: the intima, media, and adventitia. The intima, the innermost layer, is a monolayer of endothelial cells (ECs), which serve as the primary responder in the regulation of vessel tone, permeability, and interaction with circulating molecules.^2^ The media contain predominantly vascular smooth muscle cells (VSMCs), which are crucial for the regulation of vessel tone, the blood stream, and blood pressure.^3^ The adventitia, the outer layer of the artery, is composed of numerous fibroblasts and myofibroblasts, and a small number of VSMCs, adipocytes, pericytes, and inflammatory cells.^4^ Under pathological conditions, these cells coordinate together and positively participate in the progression of vascular diseases. The development of vascular diseases depends on many factors, such as inflammation,^5^, ^6^ oxidative stress,^7^, ^8^ DNA damage,^9^, ^10^ endoplasmic reticulum (ER) stress,^11^, ^12^ and metabolic reprogramming.^3^ Among these factors, inositol‐requiring protein 1α (IRE1α) plays an essential role in multiple mechanisms responsible for vascular diseases. In this review, we summarize the role of IRE1α in vascular diseases.

## 2 THE LOCALIZATION, STRUCTURE, AND SIGNALING PATHWAY OF IRE1α

To fully understand the role of IRE1α in vascular disease, we will provide a brief overview of the localization, structure, and signaling pathway of IRE1α.

### 2.1 ER stress and the unfolded protein response (UPR)

In eukaryotic cells, the ER is the largest membrane‐bound organelle and performs pivotal cellular functions such as membrane biogenesis, protein synthesis and modification, lipid synthesis and trafficking, and calcium storage and secretion.^13^, ^14^ The ER is essential for proteostasis, as more than one‐third of cellular proteins are synthesized and folded in the ER. Under a wide range of pathophysiological conditions, the accumulation of unfolded or misfolded proteins overwhelms the protein folding capability of the ER, leading to ER stress and the activation of an adaptive response called the UPR^15^ to preserve proteostasis, which includes protein overaccumulation, improved protein folding, and the degradation of misfolded proteins.^11^ Three main signaling branches mediate the UPR, known as IRE1 (α and β), activating transcription factor 6α (ATF6α) and ATF6β, and protein kinase RNA (PKR)‐like ER kinase (PERK). Under severe or consistent ER stress, homeostasis is disrupted in the ER, and overactivation of the UPR results in the activation of cell death programs.^13^, ^16^ Work over the last 3 decades has pinpointed the UPR as a driving force for the progression of a variety of diseases, including cancer,^17^, ^18^, ^19^ central nervous system diseases,^20^, ^21^ metabolic diseases,^22^, ^23^, ^24^ and CVD.^11^

### 2.2 The structure of IRE1α

**FIGURE 1:**
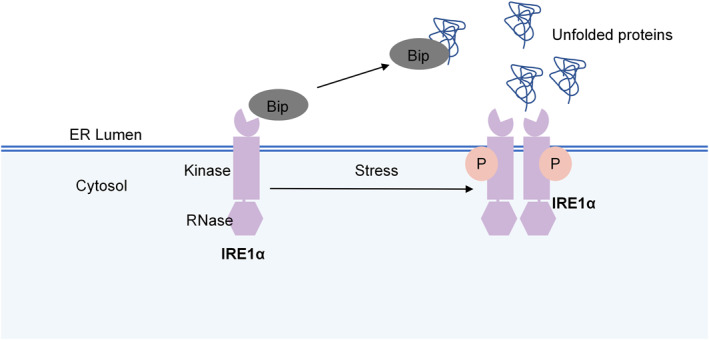
The structure of IRE1α. IRE1α consist of two domains: the endoplasmic reticulum luminal domain, and serine/threonine kinase and endoribonuclease (RNase) domains. In resting conditions, IRE1α binds to Bip/GRP78. Upon stress, IRE1α disintegrates with Bip/GRP78 and activated via autophosphorylation and dimerization/oligomerization.

IRE1 is the most ancient branch of the UPR‐related signaling pathway and is evolutionarily conserved from yeast to humans.^25^ IRE1 has two isoforms: IRE1α (encoded by the gene *ER to nucleus signaling 1 (Ern1)*) and IRE1β (encoded by the gene *Ern2*). IRE1α is abundant and prevalent in almost all tissues, while IRE1β is expressed only in airway mucous cells and intestinal epithelial cells.^26^ IRE1 consists of two domains: the N‐terminal, ER luminal domain, and the C‐terminal cytoplasmic region, serine/threonine kinase and endoribonuclease (RNase) domains (Figure 1).^27^ The LD is used to sense unfolded or misfolded proteins and monitor ER homeostasis, and the C‐terminal domain triggers the UPR.^27^ The unfolded protein can directly bind to the LD, which triggers IRE1 oligomerization and allows trans‐autophosphorylation of the kinase domain^28^. The only known substrate of the kinase is IRE1 itself, and the RNase of IRE1 is regulated by the intrinsic kinase module of IRE1.^28^

### 2.3 The activation of IRE1α

ER plays a critical role in protein and lipid synthesis, and regulate lipid and glucose metabolism.^29^ As a branch of UPR, IRE1α has been reported to be activated by several types of signals, including nutrients, metabolisms, and hormones.^13^ Glucose and lipid have been considered as stressors for activating IRE1α‐signaling pathway. It has been well‐documented that high glucose stimulated the hyperactivation and phosphorylation of IRE1α in pancreatic *β* cells,^30^, ^31^ and the prolonged activation of IRE1α contributes to *β* ‐cell apoptosis in type 1 diabetes.^32^ Moreover, high glucose activated IRE1α‐signaling pathway in retinal muller cells and renal tubular epithelial cells, which further results in diabetic retinopathy and diabetic nephropathy, respectively.^33^, ^34^ In turn, the highly phosphorylated IRE1α in the liver drives hyperglycemia under metabolic ER stress.^35^ Lipid also stimulates the activation of IRE1α. IRE1α could be activated by lipid independently of its lumenal unfolded protein stress‐sensing domain.^36^ Under metabolic ER stress conditions, activation of IRE1α underlies the dysregulation of thermogenic fat in obese mice.^37^ Besides, IRE1α has been reported to be activated in both human and rodent obese adipose tissue.^38^, ^39^ In addition, high fat diet induced the activation of IRE1α in macrophages, hepatocytes, and ECs.^40^, ^41^, ^42^, ^43^

Numerous studies have shown that IRE1α could be activated by metabolic hormones. Insulin is an anabolic hormone, which is a participant in protein synthesis and lipogenesis, and results in increased load of protein folding in the ER.^13^ The phosphorylation of IRE1α was observed in mice with hyperinsulinemia and insulin resistance induced by high fat diet.^44^ Targeting IRE1α improves insulin resistance and glucose intolerance in obese mice.^39^ Besides, mice with *β* cell‐specific deletion of IRE1α exhibited a diabetic phenotype, with less insulin secretion and elevated blood glucose level after feeding.^45^ In addition, IRE1α is metabolically activated by glucagon in the liver, and glucagon or epinephrine could stimulate protein kinase A, which further phosphorylated IRE1α at Ser^724^.^35^

### 2.4 The signaling pathways of IRE1α

**FIGURE 2:**
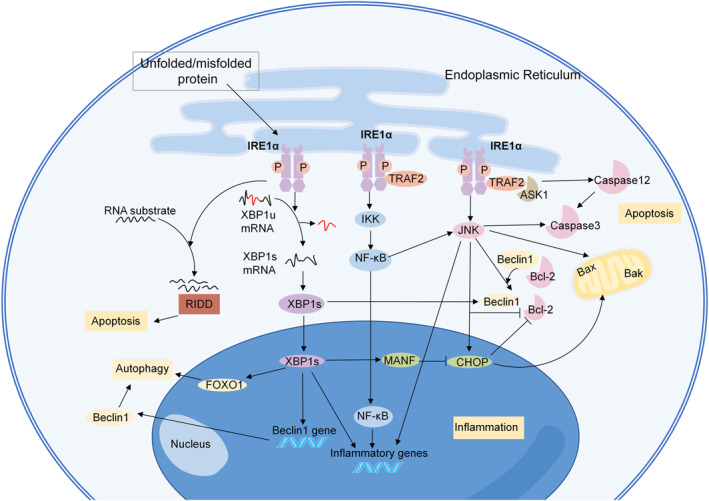
The IRE1α‐signaling pathway. The active RNase cleaves 26 nucleotides from unspliced form of XBP1 (XBP1u) mRNA and produce the spliced form of XBP1 (XBP1s) mRNA. XBP1s is a crucial transcription factor, which could translocate into the nucleus and initiate the transcriptional process. IRE1α also cleaves a subset of ER‐associated mRNAs. IRE1α activates several signaling pathways through inflammation, apoptosis, and autophagy. ASK1, apoptosis‐signaling kinase 1; CHOP, C/EBP‐homologous protein; FOXO1, Forkhead box O1; IKK, IκB kinase; JNK, c‐Jun NH2‐terminal kinase; LD, luminal domain; MANF, mesencephalic astrocyte‐derived neurotrophic factor; NF‐κB, nuclear factor‐kappa B; RIDD, regulated IRE1‐dependent decay; TRAF2, tumor necrosis factor receptor associated factor 2; XBP1s, spliced form of X‐box binding protein‐1; and XBP1u, unspliced form of X‐box binding protein‐1.

Under resting conditions, IRE1α binds to Bip/GRP78 in the ER.^24^ Upon stress, IRE1α disintegrates with Bip/GRP78 and is activated via autophosphorylation and dimerization/oligomerization, leading to RNase activation .^13^ In mammalian cells, the active RNase cleaves 26 nucleotides from the unspliced form of X‐box binding protein‐1 (XBP1u) mRNA and produces the spliced form of XBP1 (XBP1s) mRNA.^46^ XBP1u contains a nuclear C‐terminus called hydrophobic region 2 and a degradation domain, while the C‐terminus of XBP1s has a transcriptional activation domain.^46^ This difference makes XBP1s a crucial transcription factor, which could translocate into the nucleus and initiate transcriptional processes, thus participating in ER biogenesis, protein folding, and ER‐associated degradation.^24^ After being translocated to the nucleus, XBP1s binds to ER stress‐responsive element I and promotes the transcription of mesencephalic astrocyte‐derived neurotrophic factor (MANF).^47^ Inactivation of MANF contributes to the upregulation of a pro‐apoptotic component of the UPR, C/EBP‐homologous protein (CHOP).^48^ In addition, IRE1α cleaves a subset of ER‐associated mRNAs, resulting in their degradation through an activity named regulated IRE1‐dependent decay (RIDD) (Figure 2).

IRE1α is associated with well‐known risk factors for vascular diseases, such as inflammation, autophagy, and apoptosis. IRE1α has been shown to trigger inflammation in vascular diseases. The dimerization of IRE1α stimulates its protein kinase domain, and induces the binding of tumor necrosis factor receptor associated factor 2 (TRAF2) to the IRE1α complex.^49^ This thereby directly activates the apoptosis‐signaling kinase 1 (ASK1) and IκB kinase (IKK) pathways, and subsequently activates the c‐Jun NH2‐terminal kinase (JNK) and nuclear factor‐kappa B (NF‐κB) pathways.^49^, ^50^, ^51^ IKK is a crucial kinase that responds to inflammatory stimuli and can increase the activity of XBP1s.^52^ IRE1α can promote inflammatory cytokine generation through the NF‐κB pathway.^53^ IRE1α/NF‐κB can activate the JNK‐signaling pathway, and therefore induce the transcription of tumor necrosis factor (TNF)‐α, interleukin (IL)‐6, and IL‐8.^54^ IRE1α also regulates the expression of proinflammatory cytokine genes through the activation of XBP1s and glycogen synthase kinase (GSK)‐3β.^55^

IRE1α can promote both apoptosis and autophagy, which are essential processes that mediate vascular diseases.^56^ IRE1α has been reported to be the main switch between these autophagy and apoptosis.^57^ Some researchers believe that the real cell autophagic‐signaling pathway induced by ER‐stress involves IRE1α, not PERK or ATF6, as IRE1α is essential for the autophagosome formation and LC3‐II conversion.^57^, ^58^, ^59^ As we have mentioned above, upon ER‐stress, IRE1α binds to TRAF2 and ASK1, and the formation of the IRE1α‐TRAF2‐ASK1 complex activates the JNK pathway, and mediates ER stress‐induced cell autophagy.^58^, ^60^ Active JNK mediates Bcl‐2 phosphorylation and disassociates the Beclin‐1/Bcl‐2 complex, thus releasing free Beclin‐1.^61^ The IRE1α‐JNK pathway also mediates autophagic cell death through the CHOP‐signaling pathway.^62^, ^63^ The IRE1α/XBP1s pathway also regulates autophagy through Beclin‐1.^64^, ^65^ Chromatin immunoprecipitation assays revealed that XBP1s can directly bind to the promoter of Beclin‐1 at the region from nt −537 to −755.^64^ XBP1s also triggers autophagy via the Forkhead Box O1 (FoxO1) pathway, while XBP1u serves as a negative regulator because it recruits FoxO1 to the 20S proteasome and promotes FoxO1 degradation.^66^, ^67^

In the early stage of ER‐stress, the phosphorylation of Bcl‐2 mediated by JNK triggers autophagy, while sustained JNK activation induces apoptosis.^68^ The IRE1α‐TRAF2‐JNK axis enhances pro‐apoptotic Bax and Bak activity and represses anti‐apoptotic Bcl‐2 activity.^69^, ^70^, ^71^, ^72^ In addition, the TRAF2 not only facilitates the activation of caspase‐3 and promotes apoptosis through the JNK pathway^73^, ^74^ but also directly activates caspase‐12, which is specifically localized at the ER and can activate cleaved‐caspase‐3.^57^, ^73^ IRE1α can induce apoptosis through CHOP, and CHOP downregulates Bcl‐2 and Mcl‐1, and releases Bax to form pores in the mitochondrial outer membrane, which subsequently activates caspase3 and induces apoptosis.^54^, ^73^ In addition, IRE1α mediates ER stress‐induced apoptosis through the RIDD pathway.^75^

## 3 IRE1 IN VASCULAR DISEASES

### 3.1 Atherosclerosis

Atherosclerosis is the most common vascular disease worldwide.^76^, ^77^, ^78^ Chronic inflammation, imbalanced lipid metabolism, and an aberrant immune system response contribute to atherosclerosis through acting on vascular cells.^79^, ^80^ The ECs, macrophages, monocytes, and foam cells are involved in atherosclerotic lesion initiation.^81^ The risk factors for atherosclerosis, such as hyperhomocysteinaemia, oxidative stress, reactive nitrogen species, and free cholesterol accumulation in macrophages, contribute to IRE1α activation, which in turn promotes atherosclerosis.^11^

Phosphorylation of IRE1α is observed in human atherosclerotic lesions but is not detected in normal arteries.^82^ Moreover, the expression of p‐IRE1α was greater in the cores of advanced carotid atherosclerotic lesions than in those of stable aortic plaques, suggesting a key role for IRE1α in atherogenesis.^82^ This notion is also supported by the results from rodent studies. The phosphorylation of IRE1α is induced in the aortic intima and atherosclerotic lesions of *ApoE* ^*−/−*^ mice, and is significantly greater in *ApoE* ^*−/−*^ mice subjected to 5/6 nephrectomy than in controls.^71^, ^83^, ^84^ Furthermore, XBP1s, the downstream of IRE1α, was abundantly detected at branch points and in atherosclerotic lesions of *ApoE* ^*−/−*^ mice and spontaneously hypertensive rats (SHRs) fed a cholesterol‐enriched diet.^65^, ^85^, ^86^

**FIGURE 3:**
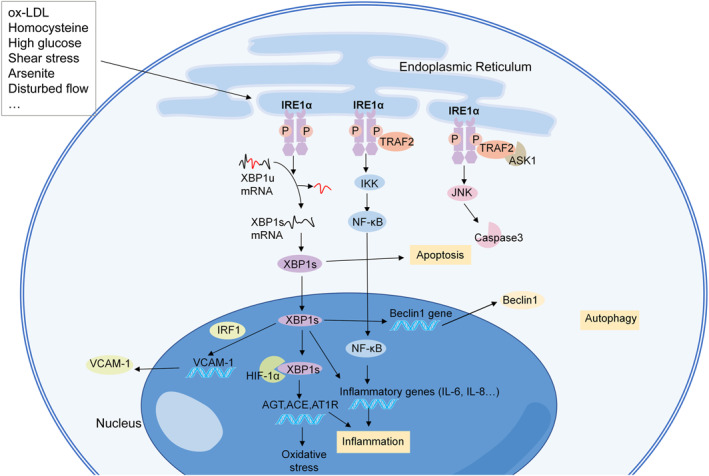
Mechanisms of IRE1α in endothelial cells (ECs) in atherosclerosis. IRE1α can be activated in ECs treated with risk factors for atherosclerosis, including oxidized low‐density lipoprotein, homocysteine, high glucose, shear stress, arsenite, and disturbed flow. IRE1α participates in the progression of atherosclerosis through inflammation, apoptosis, and autophagy. ACE, angiotensin‐converting enzyme; AGT, angiotensinogen; ASK1, apoptosis‐signaling kinase 1; AT1R, angiotensin II type 1 receptor; HIF1α, hypoxia inducible factor 1α; IRF1, interferon regulatory factor 1; IKK, IκB kinase; JNK, c‐Jun NH2‐terminal kinase; NF‐κB, nuclear factor‐kappa B; ox‐LDL, oxidized low‐density lipoprotein; TRAF2, tumor necrosis factor receptor associated factor 2; VCAM‐1, vascular cell adhesion molecule‐1; XBP1s, spliced form of X‐box binding protein‐1; and XBP1u, unspliced form of X‐box binding protein‐1.

The initiation of atherosclerosis begins with the EC dysfunction; ECs facilitate the transport of low‐density lipoprotein (LDL) into the subendothelial space, and the accumulation of LDL provokes an immune response.^87^ In the atherosclerotic model of swine, the expression of both p‐IRE1α and XBP1 was upregulated in the endothelium from the athero‐susceptible arterial region compared with the athero‐protected region.^88^ In addition, overexpression of XBP1s induces atherosclerotic lesions in a thoracic aorta isograft model.^85^ Numerous studies have reported that the activation of IRE1α in human umbilical vein ECs (HUVECs) and human aortic ECs (HAECs) is associated with risk factors for atherosclerosis, including LDL, homocysteine, and high glucose levels (Figure 3).^89^, ^90^, ^91^, ^92^ In addition, exposure to arsenite, an inducer of atherosclerosis, can activate IRE1α/XBP1s to promote hypoxia inducible factor 1α (HIF1α) accumulation.^93^ The XBP1s/HIF1α complex further regulates the transcription of angiotensinogen, angiotensin‐converting enzyme, and angiotensin II type 1 receptor, which play a role in endothelial dysfunction and inflammatory reactions.^93^ Inhibition of the IRE1α/XBP1s/HIF1α pathway attenuated arsenite‐induced oxidative stress and the proinflammatory response.^93^

EC apoptosis plays a pivotal role in the initiation and progression of atherosclerosis. IRE1α participates in the apoptosis of ECs, which promotes atherosclerotic plaque formation and destabilization.^94^ The IRE1α‐JNK pathway plays a role in the apoptotic effect in oxidized LDL (oxLDL)‐treated ECs, and inhibiting the IRE1α/JNK/caspase‐3 pathway could protect HUVECs against apoptosis.^74^, ^82^ Sustained activation of XBP1s results in HUVEC apoptosis, endothelial denudation, and lesion progression through downregulation of the VE‐cadherin and activation of multiple caspases.^85^ The IRE1α‐XBP1s pathway also regulates the autophagy of ECs in atherosclerosis and induces an autophagic response and death in ECs through the transcriptional activation of Beclin‐1.^64^ IRE1 also triggers inflammatory pathways. The IRE1α/NF‐κB pathway results in lipopolysaccharide‐induced HUVEC injury through the promotion of inflammatory cytokine production.^53^ In addition, XBP1 is an essential mediator of inflammatory factors in ECs, including IL‐6, IL‐8, monocyte chemoattractant protein (MCP)‐1, and Chemokine (CXC) motif ligand 3 (CXCL3).^95^

Atherosclerosis tends to occur in arteries with bifurcations, which exhibit turbulent blood flow, where shear stress is low.^81^, ^96^ Low shear stress regulates inflammation in HAECs through the activation of XBP1, which together with interferon regulatory factor 1, upregulates the expression of the vascular cell adhesion molecule‐1.^97^ Disturbed flow leads to the disturbances in CLOCK expression and the activation of the IRE1α‐XBP1 pathway, which results in endothelial‐to‐mesenchymal transition and inflammation, and ultimately vulnerable plaque progression.^98^ In contrast, steady laminar flow can downregulate XBP1s through the phosphoinositide 3‐kinase (PI3K)/Akt pathway.^99^

**FIGURE 4:**
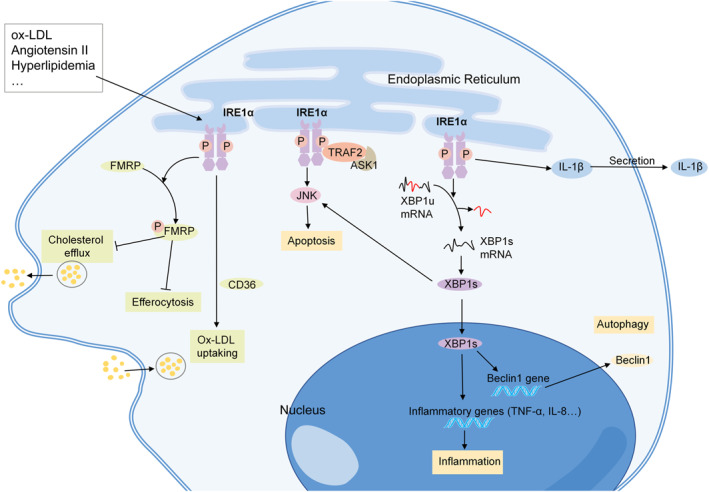
Mechanisms of IRE1α in macrophages in atherosclerosis. IRE1α can be activated in macrophages treated with oxidized low‐density lipoprotein and angiotensin II. IRE1α participates in the progression of atherosclerosis through inflammation, apoptosis, and autophagy. IRE1 kinase activation induces Fragile X Mental Retardation protein (FMRP) phosphorylation, and suppresses the macrophage cholesterol efflux and efferocytosis. IRE1α also promotes oxLDL uptaking through CD36. ASK1, apoptosis‐signaling kinase 1; FMRP, Fragile X Mental Retardation protein; JNK, c‐Jun NH2‐terminal kinase; ox‐LDL, oxidized low‐density lipoprotein; TRAF2, tumor necrosis factor receptor associated factor 2; XBP1s, spliced form of X‐box binding protein‐1; and XBP1u, unspliced form of X‐box binding protein‐1.

The macrophages in advanced atherosclerotic lesions contribute to lesion necrosis, plaque rupture, and acute atherothrombotic vascular occlusion.^71^ Transcriptome analysis revealed that IRE1α regulates the expression of proatherogenic genes in primary mouse bone marrow‐derived macrophages (BMDMs).^100^ In the peritoneal macrophages of *ApoE* ^*−/−*^ mice with hyperlipidemia, the phosphorylation of IRE1α is observed.^101^ In vitro, cholesterol can induce the expression of both p‐IRE1α and XBP1 in macrophages.^71^, ^72^, ^102^ Stimulation of macrophages with angiotensin II also induces the phosphorylation of IRE1α in a dose‐ and time‐dependent manner.^83^ In addition, XBP1 splicing is enhanced in oxLDL‐induced human leukemic monocytic THP‐1 cells and the mouse macrophage‐like cell line J774A1^103^, ^104^, ^105^ (Figure 4).

The apoptosis of macrophages is one of the main consequences of prolonged IRE1α activation. Activated IRE1α in macrophages affects the TRAF2‐ASK1‐JNK pathway, and the subsequent apoptosis.^71^, ^72^ In addition, the activation of the IRE1α‐XBP1‐JNK axis leads to the apoptosis of foam cells.^106^ As for XBP1s, Tian et al. revealed that transient overexpression of XBP1s in macrophages induces autophagy through the upregulation of Beclin‐1, and enhances cell proliferation, while sustained overexpression of XBP1s induces apoptosis, thus decreasing cell proliferation.^65^ In addition to apoptosis, many other pathways are involved in IRE1α‐mediated atherosclerosis. Recently, Yildirim reported that IRE1 kinase activation induces fragile X mental retardation protein (FMRP) phosphorylation, and suppresses the macrophage cholesterol efflux and efferocytosis.^101^ IRE1α also plays a role in macrophage‐derived foam cell formation by taking up more oxLDL, which is partly mediated by CD36.^107^ In addition, IRE1α regulates the IL‐1β secretion in lipid‐stimulated mouse BMDMs and human peripheral blood monocytes.^100^ In addition, XBP1s promotes macrophage‐mediated inflammation through TNF‐α and IL‐8 production, and foam cell accumulation.^108^

In addition to its role in ECs and macrophages, the IRE1α‐JNK pathway is also involved in oxLDL‐induced apoptosis in VSMCs.^109^ In addition, XBP1 drives the plasma cell maturation and protective antibody responses.^110^ B‐cell‐specific XBP1 deficiency increases the apoptotic cell accumulation and antibody deposition in atherosclerotic plaques in *Ldlr* ^*−/−*^ mice and increases the necrotic core area.^110^

### 3.2 Systemic hypertension and pulmonary arterial hypertension (PAH)

Currently, the role of IRE1α in hypertension remains largely unknown. In animal models, the protein levels of IRE1α, p‐IRE1α, and XBP1 are elevated in SHR arteries.^111^, ^112^, ^113^ The oxidation of IRE1α is partly regulated by nicotinamide adenine dinucleotide phosphate oxidase (Nox)‐4, and may serve as a counterregulatory mechanism against oxidative stress in hypertension.^111^ Moreover, the administration of an IRE1α inhibitor reduces the proliferation of VSMCs in SHRs.^111^

PAH is a life‐threatening disease characterized by increased pulmonary vascular resistance and vascular remodeling.^114^ In a rat model of PAH, the protein expression of IRE1α, p‐IRE1α, and XBP1s was significantly greater in the lung tissues of rats with PAH than in those without PAH,^115^, ^116^, ^117^, ^118^, ^119^ and knocking down XBP1s ameliorated PAH in rats.^115^, ^116^ Vascular remodeling is largely due to the proliferation and resistance of pulmonary artery smooth muscle cells (PASMCs) to apoptosis.^116^ The upregulation of p‐IRE1α and XBP1s is also observed in PASMCs in PAH.^120^ Recently, Jiang et al. reported that XBP1s promotes the proliferation, migration, and apoptotic resistance of PASMCs through the p‐JNK/mitogen‐activated protein kinase (MAPK) pathway.^115^ The overexpression of XBP1s also increases the expression of cell cycle‐related proteins.^120^

### 3.3 Aortic aneurysm and aortic dissection

Aneurysm is a life‐threatening vascular disease that is characterized by a permanent aortic dilatation.^121^ A study analyzing the expression of ER stress markers in abdominal aorta samples from abdominal aortic aneurysm (AAA) patients (*n* = 96) and healthy controls (*n* = 17) revealed that IRE1α/XBP1s levels are significantly greater in patients with AAA than in healthy controls, and the differences remained statistically significant after adjusting for age, sex, smoking status, hypertension status, and diabetes status.^122^ Moreover, strong immunostaining for XBP1 is observed mainly in VSMCs in the medial layer and, to a lesser extent, in lymphocytes in the inflammatory infiltration area of the aneurysmatic wall.^122^ In animal models, the expression of XBP1s is increased in AAAs in angiotensin II‐treated *ApoE* ^*−/−*^ mice.^123^ Interestingly, Zhao et al. reported that XBP1u, not XBP1s, is repressed in the early stage of aneurysm formation in angiotensin II‐infused *ApoE* ^*−/−*^ mice, which is correlated with VSMC dedifferentiation.^121^ XBP1u maintains VSMC homeostasis through FoxO4 interactions.^121^ In addition, XBP1u deficiency facilitates VSMC switching to a proinflammatory and proteolytic phenotype, and stimulates both thoracic and abdominal aortic aneurysm formation in mice.^121^ Recently, Chen et al. analyzed the functional enrichment of differentially expressed genes on the basis of Gene Expression Omnibus microarray datasets, and the results revealed that the expression of the IRE1α pathway is significantly upregulated in intracranial aneurysms and that the IRE1α pathway is highly related to FKBP14, Bax, and SEC61B expression.^124^

Aortic dissection is a rare but catastrophic disease with an extremely high mortality rate.^125^ The protein expression of p‐IRE1α and XBP1s is significantly greater in aortic specimens from patients with aortic dissection than in those from patients without aortic dissection.^125^, ^126^ Moreover, the phosphorylation of IRE1α is also increased in animal models of aortic dissection and angiotensin II‐treated rat aortic SMCs.^126^ Angiotensin II can promote the nuclear translocation of XBP1s, further resulting in the gene transcription of CHOP, cleaved caspase 3, Bax, and Bcl‐2.^125^ In addition, Shi et al. revealed that the IRE1‐XBP1‐CHOP axis accelerates the apoptosis of VSMCs in the aortic media and promotes aortic dissection.^126^

### 3.4 IRE1α is involved in the pathological process related to vascular diseases

Neointimal hyperplasia is a universal response to vascular injury and is the leading cause of vascular restenosis.^127^ The known risk factors for neointimal hyperplasia include vascular injury, systemic inflammation, diabetes, and turbulent flow.^128^ p‐IRE1α and XBP1s are increased in rat carotid artery balloon‐injured, mouse wire‐injured, and disturbed flow‐induced neointimal hyperplasia models.^129^, ^130^, ^131^ In addition, the IRE1α/XBP1s axis is involved in increased endothelial‐mesenchymal transition and subsequent carotid artery stenosis.^98^ The proliferation and migration of VSMCs play essential roles in the progression of neointimal hyperplasia. In vitro, both p‐IRE1α and XBP1s are upregulated in the platelet‐derived growth factor (PDGF)‐BB stimulated human and rodent VSMCs.^129^, ^130^, ^131^ Inhibiting the IRE1α pathway reduces the hypertrophy and proliferation of VSMCs, which is partly mediated via inhibition of the NF‐κB pathway.^132^ XBP1s increases the proliferation of VSMCs through the PI3K/Akt pathway and enhances the proliferation of VSMCs by suppressing calponin h1 and modulating the PDGF/transforming growth factor (TGF)‐β pathway.^131^ Moreover, Angbohang et al. reported that XBP1s in VSMCs upregulates type IV collagen alpha 1/2 (COL4A1/2) transcription and induces soluble COL4A1 secretion, further recruiting stem cell antigen 1‐positive‐vascular progenitor cells (VPCs) and leading to neointimal formation.^133^

Vascular calcification is considered as a pathological process associated with CVD, including atherosclerosis, hypertension, and coronary artery disease.^134^ It has been well‐documented that VSMCs play indispensable roles in the progression of vascular calcification.^3^ The phosphorylation of IRE1α and increased expression of XBP1s are observed in calcified VSMCs.^46^, ^135^ The expression of p‐IRE1α and XBP1 is increased in bone morphogenetic protein‐2 (BMP2)‐induced human coronary artery smooth muscle cells (HCSMCs).^136^ XBP1s is also upregulated in stearate‐stimulated VSMC osteoblastic differentiation and mineralization.^137^ XBP1s not only directly binds to the promoter of Runx2 in HCSMCs,^136^ but also promotes the activation of *β*‐catenin/T‐cell factor, the key regulator of vascular calcification, and facilitates Runx2 and Msx2 transcription.^46^ Overexpression of XBP1s accelerates high phosphate‐induced VSMC calcification, and inhibition of XBP1s through IRE1α siRNA alleviates VSMC calcification^29^. In addition, OxLDL promotes osteoblastic differentiation through the IRE1α/XBP1s/Runx2 pathway and activates inflammation through the IRE1α/JNK and NF‐κB pathways.^138^ The IRE1α/JNK axis is also activated in parathyroid hormone‐induced HASMCs.^139^ Recently, Yang et al. reported that XBP1u protects against calcification via an ER stress‐independent signaling pathway.^46^ The XBP1u level is lower in the radial arteries of chronic kidney disease patients with calcification than in those without calcification.^46^ Smooth muscle‐specific knockout of XBP1u aggravated 5/6 nephrectomy and adenine diet‐induced vascular calcification.^46^ Interactome analysis revealed that XBP1u binds directly to *β*‐catenin, promotes its ubiquitin‐proteasomal degradation, and inhibits Runx2 and Msx2 transcription.^46^

### 3.5 IRE1α is involved in vascular diseases mediated by common risk factors

Age is considered as an important risk factor for vascular diseases. Bioinformatic analysis of transcriptome datasets revealed that the expression of XBP1 is upregulated in aged *ApoE* ^*−/−*^ mice.^140^ The phosphorylation of IRE1α is also observed in the aortas of aged mice and aged *ApoE* ^*−/−*^ mice.^140^, ^141^ In addition, the protein levels of p‐IRE1α and XBP1s are increased in aged HUVECs.^141^, ^142^ Compared with that in young fibroblasts, the IRE1α/XBP1s axis is also activated in aged fibroblasts.^143^ In aging cells, activated Nox4 is involved in the dissociation of heat shock protein 90 from IRE1α.^142^ Additionally, IRE1α‐mediated RIDD facilitates premature senescence through the degradation of Id1 mRNA.^144^

Smoking is a leading cause of CVD. Smoking increases the risk of mostly CVD subtypes, and the risk increases with smoking intensity.^145^ Cigarette smoke extracts significantly activate the IRE1α/XBP1s axis.^146^, ^147^ The phosphorylation of IRE1α is also induced in nicotine‐induced human coronary artery ECs.^148^

Previous studies have demonstrated that increased levels of circulating advanced glycation end products (AGEs) promote vascular diseases.^149^, ^150^, ^151^ IRE1α/JNK have been reported to participate in AGE‐induced cell apoptosis.^152^, ^153^ AGEs induce the phosphorylation of IRE1α and the JNK‐signaling pathway in a time‐ and dose‐dependent manner in HUVECs.^154^ AGEs also directly induce XBP1 expression in HAECs and play a role in apoptosis, and further lead to endothelial dysfunction.^149^ IRE1α knockdown inhibits AGE‐induced NF‐κB p65 nuclear translocation and the inflammatory response.^154^, ^155^ In addition, IRE1α knockdown suppresses Bax upregulation, Bcl‐2 downregulation, and caspase‐3 activation, thereby ameliorating apoptosis.^153^

## 4 TARGETING IRE1α IN VASCULAR DISEASES

Given the role of IRE1α in the pathogenesis of vascular diseases, strategies targeting IRE1α are emerging as potential therapeutic avenues for disease intervention. Two IRE1α RNase specific inhibitors, STF‐083010 and 4μ8c, are administered in the models of atherosclerosis.^100^ STF‐083010 is an inhibitor of IRE1α RNase activity that does not affect its kinase activity and selectively inhibits XBP1 mRNA splicing.^156^ 4μ8c is also a small molecule inhibitor that selectively inhibits RNase activity of IRE1α. Compared with STF‐083010, 4μ8c also inhibits RIDD mRNA degradation.^157^ Both STF‐083010 and 4μ8c suppress lipid‐induced mitochondrial reactive oxygen species (ROS) production and inflammasome activation in macrophages and block the Th‐1 immune responses and IL‐18 cytokine release, which further reduce the plaque area and alter the plaque composition of *ApoE* ^*−/−*^ mice .^100^ In addition, the IRE1α kinase‐specific inhibitor, AMG‐18, prevented the phosphorylation of IRE1α, reduced the phosphorylation of FMRP, and significantly reduced the foam cell area and necrotic core area of atherosclerotic lesions in *ApoE* ^*−/−*^ mice.^101^ AMG‐18 also increases the efferocytosis of apoptotic cells in both peritoneal macrophages and BMDMs.^101^

In addition to the roles of the STF‐083010 and 4μ8c in atherosclerosis, their roles have also been reported in other vascular diseases. For example, 4μ8c has been reported to prevent pulmonary vascular remodeling and PAH through inhibiting the IRE1α/XBP1s pathway.^120^ The administration of 4μ8c also promotes apoptosis and inhibits the proliferation and migration of PASMCs.^118^ Moreover, 4μ8c decreases the senescence‐associated *β*‐galactosidase production in fibroblasts.^143^ In SHRs, using the compound STF‐083010 disrupts the IRE1α/XBP1s pathway and reduces the proliferation of VSMCs.^111^ In addition, STF‐083010 can block the production of proinflammatory factors and the expression of adhesion molecules in HUVECs.^158^ STF‐083010 also reduce the degree of endothelial‐mesenchymal transition in primary mouse aortic ECs exposed to disturbed flow.^98^ MKC‐3946, an IRE1α RNase domain inhibitor, was found to reverse the XBP1s‐associated ROS production, phenotype switch, and apoptosis of VSMCs, further alleviating aortic dissection.^125^ These findings above underscore the therapeutic potential of IRE1α inhibitors in the management of vascular diseases.

## 5 CHALLENGES FOR FUTURE RESEARCH

Accumulating evidence indicates that IRE1α plays an important role in the pathogenesis of vascular disease. In this context, there are many attempts to manage vascular disease by administering IRE1α inhibitors. Pharmacological modulation of IRE1α activity through targeting either the RNase domain or the kinase domain has shown potential for the management of vascular diseases. However, most of these studies were conducted ex vivo or in vitro, which could neither mimic the comprehensive environment in humans nor accurately represent the pathological changes in human disease. Therefore, clinical studies are warranted to validate the results of these studies. Clinical trials assessing these inhibitors are still lacking, and it remains to be discovered how these strategies can be applied to the clinical studies. To translate the current knowledge about the IRE1α‐signaling pathway into clinical treatment for vascular disease is still challenging. In addition, globally targeting IRE1α may induce severe adverse effects. Thus, selectively targeting IRE1α in an organ‐specific or organelle‐specific manner might be a promising way in the treatment of vascular diseases.

## 6 CONCLUSIONS

Emerging evidence suggests that the IRE1α‐signaling pathway contributes to the progression of vascular diseases, including atherosclerosis, systemic hypertension and PAH, aortic aneurysm, and aortic dissection, by mediating apoptosis and autophagy, and trigger inflammatory response. Currently, most studies on the role of IRE1α in vascular diseases originate from animal studies and in vitro studies, which largely limits our understanding of the pathological changes in human diseases. In addition, although small inhibitors targeting IRE1α have potential roles in the prevention and treatment of vascular diseases, clinical trials are still lacking. Therefore, further investigations are expected to identify the precise role of IRE1α and develop new therapeutic strategies.

## AUTHOR CONTRIBUTIONS

JS wrote the main manuscript text and prepared figures. FH and XGD revised the final version. All the authors read and approved the final manuscript.

## CONFLICT OF INTEREST STATEMENT

The authors declare no competing financial interests.

## ETHIC STATEMENT

Not applicable.

## Data Availability

Data sharing is not applicable to this article as no new data were created or analyzed in this study.

## References

[1] Roth, Gregory A. , Degu Abate , Kalkidan Hassen Abate , Solomon M. Abay , Cristiana Abbafati , Nooshin Abbasi , Hedayat Abbastabar , et al. 2018. “Global, Regional, and National Age‐sex‐specific Mortality for 282 Caus Es of Death in 195 Countries and Territories, 1980‐2017: a Systematic Analysis for the Global Burden of Disease Study 2017.” Lancet (London, England) 392(10159): 1736–1788. 10.1016/S0140-6736(18)32203-7. 30496103 PMC6227606

[2] Pi, X. , L. Xie , and C. Patterson . 2018. “Emerging Roles of Vascular Endothelium in Metabolic Homeostasis.” Circulation Research 123(4): 477–494. 10.1161/CIRCRESAHA.118.313237. 30355249 PMC6205216

[3] Shi, J. , Y. Yang , A. Cheng , G. Xu , and F. He . 2020. “Metabolism of Vascular Smooth Muscle Cells in Vascular Diseases.” American Journal of Physiology ‐ Heart and Circulatory Physiology 319(3): H613–H631. 10.1152/ajpheart.00220.2020. 32762559

[4] Tinajero, M. G. , and A. I. Gotlieb . 2020. “Recent Developments in Vascular Adventitial Pathobiology: The Dynamic Adventitia as a Complex Regulator of Vascular Disease.” American Journal Of Pathology 190(3): 520–534. 10.1016/j.ajpath.2019.10.021. 31866347

[5] Takahashi, M . 2022. “NLRP3 Inflammasome as a Key Driver of Vascular Disease.” Cardiovascular Research 118(2): 372–385. 10.1093/cvr/cvab010. 33483732

[6] Dutzmann, J. , J. M. Daniel , J. Bauersachs , D. Hilfiker‐Kleiner , and D. G. Sedding . 2015. “Emerging Translational Approaches to Target STAT3 Signalling and its Impact on Vascular Disease.” Cardiovascular Research 106(3): 365–374. 10.1093/cvr/cvv103. 25784694 PMC4431663

[7] Drummond, G. R. , S. Selemidis , K. K. Griendling , and C. G. Sobey . 2011. “Combating Oxidative Stress in Vascular Disease: NADPH Oxidases as Therapeutic Targets.” Nature Reviews Drug Discovery 10(6): 453–471. 10.1038/nrd3403. 21629295 PMC3361719

[8] Zemskov, E. A. , Q. Lu , W. Ornatowski , C. N. Klinger , Ankit A. Desai , E. Maltepe , J. X.‐J. Yuan , T. Wang , J. R. Fineman , and S. M. Black . 2019. “Biomechanical Forces and Oxidative Stress: Implications for Pulmonary Vascular Disease.” Antioxidants and Redox Signaling 31(12): 819–842. 10.1089/ars.2018.7720. 30623676 PMC6751394

[9] Sanchis, P. , C. Y. Ho , Y. Liu , L. E. Beltran , S. Ahmad , A. P. Jacob , M. Furmanik , et al. 2019. “Arterial "inflammaging" Drives Vascular Calcification in Children on Dialysis.” Kidney International 95(4): 958–972. 10.1016/j.kint.2018.12.014. 30827513 PMC6684370

[10] Uryga, A. , K. Gray , and M. Bennett . 2016. “DNA Damage and Repair in Vascular Disease.” Annual Review of Physiology 78(1): 45–66. 10.1146/annurev-physiol-021115-105127. 26442438

[11] Ren, J. , Y. Bi , J. R. Sowers , C. Hetz , and Y. Zhang . 2021. “Endoplasmic Reticulum Stress and Unfolded Protein Response in Cardiovascular Diseases.” Nature Reviews Cardiology 18(7): 499–521. 10.1038/s41569-021-00511-w. 33619348

[12] Rao, Z. , Y. Zheng , L. Xu , Z. Wang , Y. Zhou , M. Chen , N. Dong , Z. Cai , and F. Li . 2022. “Endoplasmic Reticulum Stress and Pathogenesis of Vascular Calcification.” Front Cardiovasc Med 9: 918056. 10.3389/fcvm.2022.918056. 35783850 PMC9243238

[13] Huang, S. , Y. Xing , and Y. Liu . 2019. “Emerging Roles for the ER Stress Sensor IRE1alpha in Metabolic Regulation and Disease.” Journal of Biological Chemistry 294(49): 18726–18741. 10.1074/jbc.REV119.007036. 31666338 PMC6901316

[14] Phillips, M. J. , and G. K. Voeltz . 2016. “Structure and Function of ER Membrane Contact Sites with Other Organelles.” Nature Reviews Molecular Cell Biology 17(2): 69–82. 10.1038/nrm.2015.8. 26627931 PMC5117888

[15] Bashir, S. , M. Banday , O. Qadri , A. Bashir , N. Hilal , N. i. Fatima , St. Rader , and K. M. Fazili . 2021. “The Molecular Mechanism and Functional Diversity of UPR Signaling Sensor IRE1.” Life Sciences 265: 118740. 10.1016/j.lfs.2020.118740. 33188833

[16] Hetz, C. , and F. R. Papa . 2018. “The Unfolded Protein Response and Cell Fate Control.” Molecular Cell 69(2): 169–181. 10.1016/j.molcel.2017.06.017. 29107536

[17] Hombach‐Klonisch, S. , M. Mehrpour , S. Shojaei , C. Harlos , M. Pitz , A. Hamai , K. Siemianowicz , et al. 2018. “Glioblastoma and Chemoresistance to Alkylating Agents: Involvement of Apoptosis, Autophagy, and Unfolded Protein Response.” Pharmacology & Therapeutics 184: 13–41. 10.1016/j.pharmthera.2017.10.017. 29080702

[18] Jin, Y. , and F. Saatcioglu . 2020. “Targeting the Unfolded Protein Response in Hormone‐Regulated Cancers.” Trends Cancer 6(2): 160–171. 10.1016/j.trecan.2019.12.001. 32061305

[19] Khateb, A. , and Z. A. Ronai . 2020. “Unfolded Protein Response in Leukemia: From Basic Understanding to Therapeutic Opportunities.” Trends Cancer 6(11): 960–973. 10.1016/j.trecan.2020.05.012. 32540455 PMC7721105

[20] Hetz, C. , and S. Saxena . 2017. “ER Stress and the Unfolded Protein Response in Neurodegeneration.” Nature Reviews Neurology 13(8): 477–491. 10.1038/nrneurol.2017.99. 28731040

[21] McLaughlin, T. , A. Medina , J. Perkins , M. Yera , J. J. Wang , and S. X. Zhang . 2022. “Cellular Stress Signaling and the Unfolded Protein Response in Retinal Degeneration: Mechanisms and Therapeutic Implications.” Molecular Neurodegeneration 17(1): 25. 10.1186/s13024-022-00528-w. 35346303 PMC8962104

[22] Song, M. J. , and H. Malhi . 2019. “The Unfolded Protein Response and Hepatic Lipid Metabolism in Non Alcoholic Fatty Liver Disease.” Pharmacology & Therapeutics 203: 107401. 10.1016/j.pharmthera.2019.107401. 31419516 PMC6848795

[23] Sha, H. , Y. He , L. Yang , and L. Qi . 2011. “Stressed Out about Obesity: IRE1alpha‐XBP1 in Metabolic Disorders.” Trends in Endocrinology and Metabolism 22(9): 374–381. 10.1016/j.tem.2011.05.002. 21703863 PMC3163776

[24] Wu, R. , Q.‐H. Zhang , Y.‐J. Lu , K. Ren , and G.‐H. Yi . 2015. “Involvement of the IRE1alpha‐XBP1 Pathway and XBP1s‐dependent Transcriptional Reprogramming in Metabolic Diseases.” DNA and Cell Biology 34(1): 6–18. 10.1089/dna.2014.2552. 25216212 PMC4281841

[25] Cubillos‐Ruiz, J. R. , S. E. Bettigole , and L. H. Glimcher . 2016. “Molecular Pathways: Immunosuppressive Roles of IRE1alpha‐XBP1 Signaling in Dendritic Cells of the Tumor Microenvironment.” Clinical Cancer Research 22(9): 2121–2126. 10.1158/1078-0432.CCR-15-1570. 26979393 PMC4854763

[26] Junjappa, R. P. , P. Patil , K. R. Bhattarai , H.‐R. Kim , and H.‐Jung Chae . 2018. “IRE1alpha Implications in Endoplasmic Reticulum Stress‐Mediated Development and Pathogenesis of Autoimmune Diseases.” Frontiers in Immunology 9: 1289. 10.3389/fimmu.2018.01289. 29928282 PMC5997832

[27] Siwecka, N. , W. Rozpędek‐Kamińska , A. Wawrzynkiewicz , D. Pytel , J. A. Diehl , and I. Majsterek . 2021. “The Structure, Activation and Signaling of IRE1 and its Role in Determining Cell Fate.” Biomedicines 9(2): 156. 10.3390/biomedicines9020156. 33562589 PMC7914947

[28] Ron, D. , and P. Walter . 2007. “Signal Integration in the Endoplasmic Reticulum Unfolded Protein Response.” Nature Reviews Molecular Cell Biology 8(7): 519–529. 10.1038/nrm2199. 17565364

[29] Peng, J. , C. Qin , B. Ramatchandirin , A. Pearah , S. Guo , M. Hussain , L. Yu , F. E. Wondisford , and L. He . 2022. “Activation of the Canonical ER Stress IRE1–XBP1 Pathway by Insulin Regulates Glucose and Lipid Metabolism.” Journal of Biological Chemistry 298(9): 102283. 10.1016/j.jbc.2022.102283. 35863429 PMC9396404

[30] Hassler, J. R. , D. L. Scheuner , S. Wang , J. Han , V. K. Kodali , P. Li , J. Nguyen , et al. 2015. “The IRE1α/XBP1s Pathway Is Essential for the Glucose Response and Protection of β Cells.” PLoS Biology 13(10): e1002277. 10.1371/journal.pbio.1002277. 26469762 PMC4607427

[31] Lipson, K. L. , S. G. Fonseca , S. Ishigaki , L. X. Nguyen , E. Foss , R. Bortell , A. A. Rossini , and F. Urano . 2006. “Regulation of Insulin Biosynthesis in Pancreatic Beta Cells by an Endoplasmic Reticulum‐Resident Protein Kinase IRE1.” Cell Metabolism 4(3): 245–254. 10.1016/j.cmet.2006.07.007. 16950141

[32] Yang, C. , P. diIorio , A. Jurczyk , B. O’Sullivan‐Murphy , F. Urano , and R. Bortell . 2013. “Pathological Endoplasmic Reticulum Stress Mediated by the IRE1 Pathway Contributes to Pre‐insulitic Beta Cell Apoptosis in a Virus‐Induced Rat Model of Type 1 Diabetes.” Diabetologia 56(12): 2638–2646. 10.1007/s00125-013-3044-4. 24121653 PMC4845659

[33] Sun, X. , C. Chen , H. Liu , and S. Tang . 2021. “High Glucose Induces HSP47 Expression and Promotes the Secretion of Inflammatory Factors through the IRE1α/XBP1/HIF‐1α Pathway in Retinal Müller Cells.” Experimental and Therapeutic Medicine 22(6): 1411. 10.3892/etm.2021.10847. 34676004 PMC8524763

[34] Ke, R. , Y. Wang , S. Hong , and L. Xiao . 2020. “Xiao L Endoplasmic Reticulum Stress Related Factor IRE1α Regulates TXNIP/NLRP 3‐mediated Pyroptosis in Diabetic Nephropathy.” Experimental Cell Research 396(2): 112293. 10.1016/j.yexcr.2020.112293. 32950473

[35] Mao, T. , M. Shao , Y. Qiu , J. Huang , Y. Zhang , B. Song , Q. Wang , et al. 2011. “PKA Phosphorylation Couples Hepatic Inositol‐Requiring Enzyme 1alpha T O Glucagon Signaling in Glucose Metabolism.” Proceedings of the National Academy of Sciences of the United States o f America 108(38): 15852–15857. 10.1073/pnas.1107394108. PMC3179066 21911379

[36] Volmer, R. , and D. Ron . 2015. “Lipid‐dependent Regulation of the Unfolded Protein Response.” Current Opinion in Cell Biology 33: 67–73. 10.1016/j.ceb.2014.12.002. 25543896 PMC4376399

[37] Chen, Y. , Z. Wu , S. Huang , X. Wang , S. He , L. Liu , Y. Hu , et al. 2022. “Adipocyte IRE1α Promotes PGC1α mRNA Decay and Restrains Adaptive Thermogenesis.” Nature Metabolism 4(9): 1166–1184. 10.1038/s42255-022-00631-8. 36123394

[38] Boden, G. , X. Duan , C. Homko , E. J. Molina , W. Song , O. Perez , P. Cheung , and S. Merali . 2008. “Increase in Endoplasmic Reticulum Stress–Related Proteins and Genes in Adipose Tissue of Obese, Insulin‐Resistant Individuals.” Diabetes 57(9): 2438–2444. 10.2337/db08-0604. 18567819 PMC2518495

[39] Wu, D. , V. Eeda , Z. Maria , K. Rawal , O. Herlea‐Pana , R. B. Undi , H.‐Y. Lim , and W. Wang . 2024. “Targeting IRE1α Improves Insulin Sensitivity and Thermogenesis and Sup Presses Metabolically Active Adipose Tissue Macrophages in Obesity.” bioRxiv: The Preprint Server for Biology. 10.1101/2024.07.17.603931. PMC12005715 40244655

[40] Sozen, E. , T. Demirel‐Yalciner , D. Sari , and N. K. Ozer . 2022. “Cholesterol Accumulation in Hepatocytes Mediates IRE1/p38 Branch of Endoplasmic Reticulum Stress to Promote Nonalcoholic Steatohepatitis.” Free Radical Biology and Medicine 191: 1–7. 10.1016/j.freeradbiomed.2022.08.024. 35995397

[41] Shen, Y. , W. Zhao , Ó. Monroig , Y. Bao , T. Zhu , L. Jiao , P. Sun , D. R. Tocher , Q. Zhou , and M. Jin . 2023. “High‐fat‐diet Induced Inflammation and Apoptosis via Activation of Ire1α in Liver and Hepatocytes of Black Seabream (Acanthopagrus Schlegelii).” Fish & Shellfish Immunology 143: 109212. 10.1016/j.fsi.2023.109212. 37926203

[42] Shan, B. , X. Wang , Y. Wu , C. Xu , Z. Xia , J. Dai , M. Shao , et al. 2017. “The Metabolic ER Stress Sensor IRE1alpha Suppresses Alternative Activation of Macrophages and Impairs Energy Expenditure in Obesity.” Nature Immunology 18(5): 519–529. 10.1038/ni.3709. 28346409

[43] Wen, Z. , X. He , J. Wang , H. Wang , T. Li , S. Wen , Z. Ren , et al. 2023. “Hyperlipidemia Induces Proinflammatory Responses by Activating STING Pathway through IRE1α‐XBP1 in Retinal Endothelial Cells.” The Journal of Nutritional Biochemistry 112: 109213. 10.1016/j.jnutbio.2022.109213. 36370931

[44] Ning, J. , T. Hong , A. Ward , J. Pi , Z. Liu , H.‐Y. Liu , and W. Cao . 2011. “Constitutive Role for IRE1α‐XBP1 Signaling Pathway in the Insulin‐Mediated Hepatic Lipogenic Program.” Endocrinology 152(6): 2247–2255. 10.1210/en.2010-1036. 21447637 PMC3100623

[45] Sedwick, C . 2015. “IRE1α Stands Astride Many Paths to Insulin Production.” PLoS Biology 13(10): e1002278. 10.1371/journal.pbio.1002278. 26469794 PMC4607496

[46] Yang, L. , R. Dai , H. Wu , Z. Cai , N. Xie , X. Zhang , Y. Shen , et al. 2022. “Unspliced XBP1 Counteracts Beta‐Catenin to Inhibit Vascular Calcification.” Circulation Research 130(2): 213–229. 10.1161/CIRCRESAHA.121.319745. 34870453

[47] Wang, D. , C. Hou , Y. Cao , Q. Cheng , L. Zhang , H. Li , L. Feng , and Y. Shen . 2018. “XBP1 Activation Enhances MANF Expression via Binding to Endoplasmic Reticulum Stress Response Elements within MANF Promoter Region in Hepatitis B.” International Journal of Biochemistry &amp; Cell Biology 99: 140–146. 10.1016/j.biocel.2018.04.007. 29649564

[48] Herranen, A. , K. Ikäheimo , T. Lankinen , E. Pakarinen , B. Fritzsch , M. Saarma , M. Lindahl , and U. Pirvola . 2020. “Deficiency of the ER‐Stress‐Regulator MANF Triggers Progressive Outer Hair Cell Death and Hearing Loss.” Cell Death & Disease 11(2): 100. 10.1038/s41419-020-2286-6. 32029702 PMC7005028

[49] Salminen, A. , K. Kaarniranta , and A. Kauppinen . 2020. “ER Stress Activates Immunosuppressive Network: Implications for Aging and Alzheimer's Disease.” Journal of Molecular Medicine (Berlin) 98(5): 633–650. 10.1007/s00109-020-01904-z. PMC7220864 32279085

[50] Adolph, T.‐E. , Lu. Niederreiter , R. S. Blumberg , and A. Kaser . 2012. “Endoplasmic Reticulum Stress and Inflammation.” Digestive Diseases 30(4): 341–346. 10.1159/000338121. 22796794 PMC3423328

[51] Schmitz, M. L. , M. S. Shaban , B. V. Albert , A. Gökçen , and M. Kracht . 2018. “The Crosstalk of Endoplasmic Reticulum (ER) Stress Pathways with NF‐kappaB: Complex Mechanisms Relevant for Cancer, Inflammation and Infection.” Biomedicines 6(2): 58. 10.3390/biomedicines6020058. 29772680 PMC6027367

[52] Liu, J. , D. Ibi , K. Taniguchi , J. Lee , H. Herrema , B. Akosman , P. Mucka , et al. 2016. “Inflammation Improves Glucose Homeostasis through IKKbeta‐XBP1s Interaction.” Cell 167(4): 1052–1066.e18. 10.1016/j.cell.2016.10.015. 27814504 PMC5908236

[53] Chen, J. , M. Zhang , M. Zhu , J. Gu , J. Song , L. Cui , D. Liu , Q. Ning , X. Jia , and L. Feng . 2018. “Paeoniflorin Prevents Endoplasmic Reticulum Stress‐Associated Inflammation in Lipopolysaccharide‐Stimulated Human Umbilical Vein Endothelial Cells via the IRE1alpha/NF‐kappaB Signaling Pathway.” Food & Function 9(4): 2386–2397. 10.1039/c7fo01406f. 29594285

[54] Li, Y. , W. Jiang , Q. Niu , Y. Sun , C. Meng , L. Tan , C. Song , X. Qiu , Y. Liao , and C. Ding . 2019. “eIF2alpha‐CHOP‐BCl‐2/JNK and IRE1alpha‐XBP1/JNK Signaling Promote Apoptosis and Inflammation and Support the Proliferation of Newcastle Disease Virus.” Cell Death & Disease 10(12): 891. 10.1038/s41419-019-2128-6. 31767828 PMC6877643

[55] Kim, S. , Y. Joe , H. J. Kim , Y.‐S. Kim , S. O. Jeong , H.‐O. Pae , S. W. Ryter , Y.‐J. Surh , and H. T. Chung . 2015. “Endoplasmic Reticulum Stress‐Induced IRE1alpha Activation Mediates Cross‐Talk of GSK‐3beta and XBP‐1 to Regulate Inflammatory Cytokine Production.” Journal of Immunology 194(9): 4498–4506. 10.4049/jimmunol.1401399. PMC4400814 25821218

[56] De Meyer, G. R. Y. , M. O. J. Grootaert , C. F. Michiels , A. Kurdi , D. M. Schrijvers , and W. Martinet . 2015. “Autophagy in Vascular Disease.” Circulation Research 116(3): 468–479. 10.1161/CIRCRESAHA.116.303804. 25634970

[57] Moretti, L. , Y. I. Cha , K. J. Niermann , and B. Lu . 2007. “Switch between Apoptosis and Autophagy: Radiation‐Induced Endoplasmic Reticulum Stress?” Cell Cycle 6(7): 793–798. 10.4161/cc.6.7.4036. 17377498

[58] Qi, Z. , and L. Chen . 2019. “Endoplasmic Reticulum Stress and Autophagy.” Advances in Experimental Medicine and Biology 1206: 167–177. 10.1007/978-981-15-0602-4_8. 31776985

[59] Chaurasia, M. , S. Gupta , A. Das , B. S. Dwarakanath , A. Simonsen , and K. Sharma . 2019. “Radiation Induces EIF2AK3/PERK and ERN1/IRE1 Mediated Pro‐survival Autophagy.” Autophagy 15(8): 1391–1406. 10.1080/15548627.2019.1582973. 30773986 PMC6613886

[60] Yu, Y. , D. Wu , Y. Li , H. Qiao , and Z. Shan . 2021. “Ketamine Enhances Autophagy and Endoplasmic Reticulum Stress in Rats and SV‐HUC‐1 Cells via Activating IRE1‐TRAF2‐ASK1‐JNK Pathway.” Cell Cycle 20(18): 1907–1922. 10.1080/15384101.2021.1966199. 34427546 PMC8525958

[61] Song, S. , J. Tan , Y. Miao , and Q. Zhang . 2018. “Crosstalk of ER Stress‐Mediated Autophagy and ER‐Phagy: Involvement of UPR and the Core Autophagy Machinery.” Journal of Cellular Physiology 233(5): 3867–3874. 10.1002/jcp.26137. 28777470

[62] Kim, T. W. , S. Y. Lee , M. Kim , C. Cheon , and S.‐G. Ko . 2018. “Kaempferol Induces Autophagic Cell Death via IRE1‐JNK‐CHOP Pathway and Inhibition of G9a in Gastric Cancer Cells.” Cell Death & Disease 9: 875. 10.1038/s41419-018-0930-1. 30158521 PMC6115440

[63] Jian, Z. , Y. Han , W. Zhang , C. Li , W. Guo , X. Feng , B. Li , and H. Li . 2022. “Anti‐tumor Effects of Dual PI3K‐HDAC Inhibitor CUDC‐907 on Activation of ROS‐IRE1alpha‐JNK‐Mediated Cytotoxic Autophagy in Esophageal Cancer.” Cell & Bioscience 12(1): 135. 10.1186/s13578-022-00855-x. 35989326 PMC9394063

[64] Margariti, A. , H. Li , T. Chen , D. Martin , G. Vizcay‐Barrena , S. Alam , E. Karamariti , et al. 2013. “XBP1 mRNA Splicing Triggers an Autophagic Response in Endothelial Cells through BECLIN‐1 Transcriptional Activation.” Journal of Biological Chemistry 288(2): 859–872. 10.1074/jbc.M112.412783. 23184933 PMC3543035

[65] Tian, P.‐G. , Z.‐X. Jiang , J.‐H. Li , Z. Zhou , and Q.‐H. Zhang . 2015. “Spliced XBP1 Promotes Macrophage Survival and Autophagy by Interacting with Beclin‐1.” Biochemical and Biophysical Research Communications 463(4): 518–523. 10.1016/j.bbrc.2015.05.061. 26026678

[66] Kishino, A. , K. Hayashi , C. Hidai , T. Masuda , Y. Nomura , and T. Oshima . 2017. “XBP1‐FoxO1 Interaction Regulates ER Stress‐Induced Autophagy in Auditory Cells.” Scientific Reports 7(1): 4442. 10.1038/s41598-017-02960-1. 28667325 PMC5493624

[67] Zhao, Y. , X. Li , M.‐Y. Cai , K. Ma , J. Yang , J. Zhou , W. Fu , et al. 2013. “XBP‐1u Suppresses Autophagy by Promoting the Degradation of FoxO1 in Cancer Cells.” Cell Research 23(4): 491–507. 10.1038/cr.2013.2. 23277279 PMC3616429

[68] Song, S. , J. Tan , Y. Miao , M. Li , and Q. Zhang . 2017. “Crosstalk of Autophagy and Apoptosis: Involvement of the Dual Role of Autophagy under ER Stress.” Journal of Cellular Physiology 232(11): 2977–2984. 10.1002/jcp.25785. 28067409

[69] Huang, R. , Z. Hui , S. Wei , D. Li , W. Li , W. Daping , and M. Alahdal . 2022. “IRE1 Signaling Regulates Chondrocyte Apoptosis and Death Fate in the Osteoarthritis.” Journal of Cellular Physiology 237(1): 118–127. 10.1002/jcp.30537. 34297411 PMC9291116

[70] Cubillos‐Ruiz, J. R. , S. E. Bettigole , and L. H. Glimcher . 2017. “Tumorigenic and Immunosuppressive Effects of Endoplasmic Reticulum Stress in Cancer.” Cell 168(4): 692–706. 10.1016/j.cell.2016.12.004. 28187289 PMC5333759

[71] Li, F. , Y. Guo , S. Sun , X. Jiang , B. Tang , Q. Wang , and L. Wang . 2008. “Free Cholesterol‐Induced Macrophage Apoptosis Is Mediated by Inositol‐Requiring Enzyme 1 Alpha‐Regulated Activation of Jun N‐Terminal Kinase.” Acta Biochimica et Biophysica Sinica 40(3): 226–234. 10.1111/j.1745-7270.2008.00396.x. 18330477

[72] Park, S.‐H. , M.‐K. Kang , Y.‐J. Choi , Y.‐H. Kim , L. D. Antika , S. S. Lim , and Y.‐H. Kang . 2016. “Dietary Compound Alpha‐Asarone Alleviates ER Stress‐Mediated Apoptosis in 7beta‐Hydroxycholesterol‐Challenged Macrophages.” Molecular Nutrition & Food Research 60(5): 1033–1047. 10.1002/mnfr.201500750. 26893256

[73] Yao, W. , X. Yang , J. Zhu , B. Gao , H. Shi , and L. Xu . 2018. “IRE1alpha siRNA Relieves Endoplasmic Reticulum Stress‐Induced Apoptosis and Alleviates Diabetic Peripheral Neuropathy In Vivo and In Vitro.” Scientific Reports 8(1): 2579. 10.1038/s41598-018-20950-9. 29416111 PMC5803253

[74] Wu, L. , X. Y. Liu , L. Wang , Y. Wang , L. Wang , B. Guan , Z. Chen , and L. Liu . 2017. “Exendin‐4 Protects HUVECs from Tunicamycin‐Induced Apoptosis via Inhibiting the IRE1a/JNK/caspase‐3 Pathway.” Endocrine 55(3): 764–772. 10.1007/s12020-016-1190-4. 27915415

[75] Go, D. , J. Lee , J.‐A. Choi , S.‐N. Cho , S.‐H. Kim , S.‐H. Son , and C.‐H. Song . 2019. “Reactive Oxygen Species‐Mediated Endoplasmic Reticulum Stress Response Induces Apoptosis of Mycobacterium Avium‐Infected Macrophages by Activating Regulated IRE1‐dependent Decay Pathway.” Cellular Microbiology 21(12): e13094. 10.1111/cmi.13094. 31386788 PMC6899680

[76] Libby, P. , J. E. Buring , L. Badimon , G. K. Hansson , J. Deanfield , M. S. Bittencourt , L. Tokgözoğlu , and E. F. Lewis . 2019. “Atherosclerosis.” Nature Reviews Disease Primers 5(1): 56. 10.1038/s41572-019-0106-z. 31420554

[77] Gisterå, A. , and G. K. Hansson . 2017. “The Immunology of Atherosclerosis.” Nature Reviews Nephrology 13(6): 368–380. 10.1038/nrneph.2017.51. 28392564

[78] Miller, C. L. , A. R. Kontorovich , K. Hao , L. Ma , C. Iyegbe , J. L. M. Björkegren , and J. C. Kovacic . 2021. “Precision Medicine Approaches to Vascular Disease: JACC Focus Seminar 2/5.” Journal of the American College of Cardiology 77(20): 2531–2550. 10.1016/j.jacc.2021.04.001. 34016266 PMC8916012

[79] Jain, T. , E. A. Nikolopoulou , Q. Xu , and A. Qu . 2018. “Hypoxia Inducible Factor as a Therapeutic Target for Atherosclerosis.” Pharmacology & Therapeutics 183: 22–33. 10.1016/j.pharmthera.2017.09.003. 28942242

[80] Solanki, A. , L. K. Bhatt , and T. P. Johnston . 2018. “Evolving Targets for the Treatment of Atherosclerosis.” Pharmacology & Therapeutics 187: 1–12. 10.1016/j.pharmthera.2018.02.002. 29414673

[81] Björkegren, J. L. M. , and A. J. Lusis . 2022. “Atherosclerosis: Recent Developments.” Cell 185(10): 1630–1645. 10.1016/j.cell.2022.04.004. 35504280 PMC9119695

[82] Sanson, M. , N. Auge , C. Vindis , C. Muller , Y. Bando , J. C. Thiers , M. A. Marachet , et al. 2009. “Oxidized Low‐Density Lipoproteins Trigger Endoplasmic Reticulum Stress in Vascular Cells: Prevention by Oxygen‐Regulated Protein 150 Expression.” Circulation Research 104: 328–336. 10.1161/CIRCRESAHA.108.183749. 19106412

[83] Yang, J. , X. Zhang , X. Yu , W. Tang , and H. Gan . 2017. “Renin‐angiotensin System Activation Accelerates Atherosclerosis in Experimental Renal Failure by Promoting Endoplasmic Reticulum Stress‐Related Inflammation.” International Journal of Molecular Medicine 39(3): 613–621. 10.3892/ijmm.2017.2856. 28098884 PMC5360357

[84] Geng, J. , H. Xu , W. Fu , X. Yu , G. Xu , H. Cao , G. Lin , and D. Sui . 2020. “Rosuvastatin Protects against Endothelial Cell Apoptosis In Vitro and Alleviates Atherosclerosis in ApoE(‐/‐) Mice by Suppressing Endoplasmic Reticulum Stress.” Experimental and Therapeutic Medicine 20(1): 550–560. 10.3892/etm.2020.8733. 32537013 PMC7282009

[85] Zeng, L. , A. Zampetaki , A. Margariti , A. E. Pepe , S. Alam , D. Martin , Q. Xiao , et al. 2009. “Sustained Activation of XBP1 Splicing Leads to Endothelial Apoptosis and Atherosclerosis Development in Response to Disturbed Flow.” Proceedings of the National Academy of Sciences of the U S A 106(20): 8326–8331. 10.1073/pnas.0903197106. PMC2676169 19416856

[86] Tumanovska, L. V. , R. J. Swanson , Z. O. Serebrovska , G. V. Portnichenko , S. V. Goncharov , B. A. Kysilov , O. O. Moibenko , and V. E. Dosenko . 2019. “Cholesterol Enriched Diet Suppresses ATF6 and PERK and Upregulates the IRE1 Pathways of the Unfolded Protein Response in Spontaneously Hypertensive Rats: Relevance to Pathophysiology of Atherosclerosis in the Setting of Hypertension.” Pathophysiology 26(3–4): 219–226. 10.1016/j.pathophys.2019.05.005. 31202527

[87] Botts, S. R. , J. E. Fish , and K. L. Howe . 2021. “Dysfunctional Vascular Endothelium as a Driver of Atherosclerosis: Emerging Insights into Pathogenesis and Treatment.” Frontiers in Pharmacology 12: 787541. 10.3389/fphar.2021.787541. 35002720 PMC8727904

[88] Civelek, M. , E. Manduchi , R. J. Riley , C. J. Stoeckert, Jr , and P. F. Davies . 2009. “Chronic Endoplasmic Reticulum Stress Activates Unfolded Protein Response in Arterial Endothelium in Regions of Susceptibility to Atherosclerosis.” Circulation Research 105(5): 453–461. 10.1161/CIRCRESAHA.109.203711. 19661457 PMC2746924

[89] Hu, H. , C. Wang , Y. Jin , Q. Meng , Q. Liu , K. Liu , and H. Sun . 2016. “Alpha‐lipoic Acid Defends Homocysteine‐Induced Endoplasmic Reticulum and Oxidative Stress in HAECs.” Biomedicine & Pharmacotherapy 80: 63–72. 10.1016/j.biopha.2016.02.022. 27133040

[90] Zhu, L. , F. Jia , J. Wei , Y. Yu , T. Yu , Y. Wang , J. Sun , and G. Luo . 2017. “Salidroside Protects against Homocysteine‐Induced Injury in Human Umbilical Vein Endothelial Cells via the Regulation of Endoplasmic Reticulum Stress.” Cardiovasc Ther 35(1): 33–39. 10.1111/1755-5922.12234. 27809414

[91] Hu, H. , C. Wang , Y. Jin , Q. Meng , Q. Liu , Z. Liu , K. Liu , X. Liu , and H. Sun . 2019. “Catalpol Inhibits Homocysteine‐Induced Oxidation and Inflammation via Inhibiting Nox4/NF‐kappaB and GRP78/PERK Pathways in Human Aorta Endothelial Cells.” Inflammation 42(1): 64–80. 10.1007/s10753-018-0873-9. 30315526 PMC6394570

[92] Xu, J. Z. , Y. L. Chai , and Y. L. Zhang . 2016. “Effect of Rosuvastatin on High Glucose‐Induced Endoplasmic Reticulum Stress in Human Umbilical Vein Endothelial Cells.” Genetics and Molecular Research 15(4). 10.4238/gmr15048935. 27813604

[93] Xu, X. , S. Liu , H. Wang , M. Hu , C. Xing , and L. Song . 2017. “Arsenite Induces Vascular Endothelial Cell Dysfunction by Activating IRE1alpha/XBP1s/HIF1alpha‐dependent ANGII Signaling.” Toxicological Sciences 160(2): 315–328. 10.1093/toxsci/kfx184. 28973481

[94] Di, M. , L. Wang , M. Li , Y. Zhang , X. Liu , R. Zeng , H. Wang , et al. 2017. “Dickkopf1 Destabilizes Atherosclerotic Plaques and Promotes Plaque Formation by Inducing Apoptosis of Endothelial Cells through Activation of ER Stress.” Cell Death & Disease 8(7): e2917. 10.1038/cddis.2017.277. 28703797 PMC5550842

[95] Gargalovic, P. S. , N. M. Gharavi , M. J. Clark , J. Pagnon , W.‐P. Yang , A. He , A. Truong , et al. 2006. “The Unfolded Protein Response Is an Important Regulator of Inflammatory Genes in Endothelial Cells.” Arteriosclerosis, Thrombosis, and Vascular Biology 26(11): 2490–2496. 10.1161/01.ATV.0000242903.41158.a1. 16931790

[96] Moerman, A. M. , S. Korteland , K. Dilba , K. van Gaalen , D. H. J. Poot , A. van Der Lugt , H. J. M. Verhagen , et al. 2021. “The Correlation between Wall Shear Stress and Plaque Composition in Advanced Human Carotid Atherosclerosis.” Frontiers in Bioengineering and Biotechnology 9: 828577. 10.3389/fbioe.2021.828577. 35155418 PMC8831262

[97] Bailey, K. A. , E. Moreno , F. G. Haj , S. I. Simon , and A. G. Passerini . 2019. “Mechanoregulation of P38 Activity Enhances Endoplasmic Reticulum Stress‐Mediated Inflammation by Arterial Endothelium.” The FASEB Journal 33(11): 12888–12899. 10.1096/fj.201900236R. 31499005 PMC6902662

[98] Tang, H. , S. Xue , G. Zhao , C. Fang , L. Cai , Z. Shi , W. Fu , et al. 2020. “CLOCK Disruption Aggravates Carotid Artery Stenosis through Endoplasmic Reticulum Stress‐Induced Endothelial‐Mesenchymal Transition.” Am J Transl Res 12: 7885–7898. 33437367 PMC7791501

[99] Kim, S. , and C. H. Woo . 2018. “Laminar Flow Inhibits ER Stress‐Induced Endothelial Apoptosis through PI3K/Akt‐dependent Signaling Pathway.” Molecular Cell 41: 964–970. 10.14348/molcells.2018.0111. PMC6277562 30396238

[100] Tufanli, O. , P. Telkoparan Akillilar , D. Acosta‐Alvear , B. Kocaturk , U. I. Onat , S. M. Hamid , I. Çimen , P. Walter , C. Weber , and E. Erbay . 2017. “Targeting IRE1 with Small Molecules Counteracts Progression of Atherosclerosis.” Proceedings of the National Academy of Sciences of the U S A 114(8): E1395–E1404. 10.1073/pnas.1621188114. PMC5338400 28137856

[101] Yildirim, Z. , S. Baboo , S. M. Hamid , A. E. Dogan , O. Tufanli , S. Robichaud , C. Emerton , et al. 2022. “Intercepting IRE1 Kinase‐FMRP Signaling Prevents Atherosclerosis Progression.” EMBO Molecular Medicine 14(4): e15344. 10.15252/emmm.202115344. 35191199 PMC8988208

[102] Shen, L. , Z. Sun , S. Chu , Z. Cai , P. Nie , C. Wu , R. Yuan , L. Hu , and B. He . 2017. “Xuezhikang, an Extract from Red Yeast Rice, Attenuates Vulnerable Plaque Progression by Suppressing Endoplasmic Reticulum Stress‐Mediated Apoptosis and Inflammation.” PLoS One 12(11): e0188841. 10.1371/journal.pone.0188841. 29190732 PMC5708751

[103] Park, S.‐H. , D. Shin , S. S. Lim , J.‐Y. Lee , and Y.‐H. Kang . 2014. “Purple Perilla Extracts Allay ER Stress in Lipid‐Laden Macrophages.” PLoS One 9(10): e110581. 10.1371/journal.pone.0110581. 25333946 PMC4198214

[104] Sanda, G. M. , M. Deleanu , L. Toma , C. S. Stancu , M. Simionescu , and A. V. Sima . 2017. “Oxidized LDL‐Exposed Human Macrophages Display Increased MMP‐9 Expression and Secretion Mediated by Endoplasmic Reticulum Stress.” Journal of Cellular Biochemistry 118(4): 661–669. 10.1002/jcb.25637. 27341688

[105] Bhansali, S. , S. Khatri , and V. Dhawan . 2019. “Terminalia Arjuna Bark Extract Impedes Foam Cell Formation and Promotes Apoptosis in Ox‐LDL‐Stimulated Macrophages by Enhancing UPR‐CHOP Pathway.” Lipids in Health and Disease 18(1): 195. 10.1186/s12944-019-1119-z. 31706299 PMC6842518

[106] Dai, M.‐X. , X.‐H. Zheng , J. Yu , T. Yin , M.‐J. Ma , L. Zhang , M. Liu , et al. 2014. “The Impact of Intermittent and Repetitive Cold Stress Exposure on Endoplasmic Reticulum Stress and Instability of Atherosclerotic Plaques.” Cellular Physiology and Biochemistry 34(2): 393–404. 10.1159/000363008. 25059288

[107] Yao, S. , C. Miao , H. Tian , H. Sang , N. Yang , P. Jiao , J. Han , C. Zong , and S. Qin . 2014. “Endoplasmic Reticulum Stress Promotes Macrophage‐Derived Foam Cell Formation by Up‐Regulating Cluster of Differentiation 36 (CD36) Expression.” Journal of Biological Chemistry 289(7): 4032–4042. 10.1074/jbc.M113.524512. 24366867 PMC3924270

[108] Yang, P. , and P. B. Yu . 2022. “A New Link in the Chain: Unspliced XBP1 in Wnt Signaling and Vascular Calcification.” Circulation Research 130(2): 230–233. 10.1161/CIRCRESAHA.121.320599. 35050688 PMC9125400

[109] Larroque‐Cardoso, P. , A. Swiader , C. Ingueneau , A. Nègre‐Salvayre , M. Elbaz , M. E. Reyland , R. Salvayre , and C. Vindis . 2013. “Role of Protein Kinase C Delta in ER Stress and Apoptosis Induced by Oxidized LDL in Human Vascular Smooth Muscle Cells.” Cell Death & Disease 4(2): e520. 10.1038/cddis.2013.47. 23449456 PMC3734829

[110] Sage, A. P. , M. Nus , J. Bagchi Chakraborty , D. Tsiantoulas , S. A. Newland , A. J. Finigan , L. Masters , C. J. Binder , and Z. Mallat . 2017. “X‐box Binding Protein‐1 Dependent Plasma Cell Responses Limit the Development of Atherosclerosis.” Circulation Research 121(3): 270–281. 10.1161/CIRCRESAHA.117.310884. 28620068

[111] Camargo, L. L. , A. P. Harvey , F. J. Rios , S. Tsiropoulou , R. N. O. Da Silva , Z. Cao , D. Graham , et al. 2018. “Vascular Nox (NADPH Oxidase) Compartmentalization, Protein Hyperoxidation, and Endoplasmic Reticulum Stress Response in Hypertension.” Hypertension 72(1): 235–246. 10.1161/HYPERTENSIONAHA.118.10824. 29844144 PMC6004120

[112] Choi, S.‐K. , M. Lim , S.‐H. Byeon , and Y.‐H. Lee . 2016. “Inhibition of Endoplasmic Reticulum Stress Improves Coronary Artery Function in the Spontaneously Hypertensive Rats.” Scientific Reports 6(1): 31925. 10.1038/srep31925. 27550383 PMC4994042

[113] Liu, L. , J. Liu , Z. Huang , X. Yu , X. Zhang , D. Dou , and Y. Huang . 2015. “Berberine Improves Endothelial Function by Inhibiting Endoplasmic Reticulum Stress in the Carotid Arteries of Spontaneously Hypertensive Rats.” Biochemical and Biophysical Research Communications 458(4): 796–801. 10.1016/j.bbrc.2015.02.028. 25686503

[114] Docherty, C. K. , K. Y. Harvey , K. M. Mair , S. Griffin , N. Denver , and M. R. MacLean . 2018. “The Role of Sex in the Pathophysiology of Pulmonary Hypertension.” Advances in Experimental Medicine and Biology 1065: 511–528. 10.1007/978-3-319-77932-4_31. 30051404

[115] Jiang, H. , Y. Niu , Y. He , X. Li , Y. Xu , and X. Liu . 2022. “Proteomic Analysis Reveals that Xbp1s Promotes Hypoxic Pulmonary Hypertension through the P‐JNK MAPK Pathway.” Journal of Cellular Physiology 237(3): 1948–1963. 10.1002/jcp.30664. 34964131

[116] Jiang, H. , D. Ding , Y. He , X. Li , Y. Xu , and X. Liu . 2021. “Xbp1s‐Ddit3 Promotes MCT‐Induced Pulmonary Hypertension.” Clinical Science 135(21): 2467–2481. 10.1042/CS20210612. 34676402 PMC8564003

[117] Pu, X. , X. Lin , X. Duan , J. Wang , J. Shang , H. Yun , and Z. Chen . 2020. “Oxidative and Endoplasmic Reticulum Stress Responses to Chronic High‐Altitude Exposure during the Development of High‐Altitude Pulmonary Hypertension.” High Altitude Medicine & Biology 21(4): 378–387. 10.1089/ham.2019.0143. 33090046

[118] Cao, X. , Y. He , X. Li , Y. Xu , and X. Liu . 2019. “The IRE1alpha‐XBP1 Pathway Function in Hypoxia‐Induced Pulmonary Vascular Remodeling, Is Upregulated by Quercetin, Inhibits Apoptosis and Partially Reverses the Effect of Quercetin in PASMCs.” Am J Transl Res 11: 641–654. 30899368 PMC6413268

[119] Wang, J.‐J. , X.‐R. Zuo , J. Xu , J.‐Y. Zhou , H. Kong , X.‐N. Zeng , W.‐P. Xie , and Q. Cao . 2016. “Evaluation and Treatment of Endoplasmic Reticulum (ER) Stress in Right Ventricular Dysfunction during Monocrotaline‐Induced Rat Pulmonary Arterial Hypertension.” Cardiovascular Drugs and Therapy 30(6): 587–598. 10.1007/s10557-016-6702-1. 27844183

[120] Yu, W. , G. Xu , H. Chen , L. Xiao , G. Liu , P. Hu , S. Li , V. Kasim , C. Zeng , and X. Tong . 2022. “The Substitution of SERCA2 Redox Cysteine 674 Promotes Pulmonary Vascular Remodeling by Activating IRE1α/XBP1s Pathway.” Acta Pharmaceutica Sinica B 12(5): 2315–2329. 10.1016/j.apsb.2021.12.025. 35646520 PMC9136575

[121] Zhao, G. , Y. Fu , Z. Cai , F. Yu , Z. Gong , R. Dai , Y. Hu , L. Zeng , Q. Xu , and W. Kong . 2017. “Unspliced XBP1 Confers VSMC Homeostasis and Prevents Aortic Aneurysm Formation via FoxO4 Interaction.” Circulation Research 121(12): 1331–1345. 10.1161/CIRCRESAHA.117.311450. 29089350

[122] Navas‐Madroñal, M. , C. Rodriguez , M. Kassan , J. Fité , J. R. Escudero , L. Cañes , J. Martínez‐González , M. Camacho , and M. Galán . 2019. “Enhanced Endoplasmic Reticulum and Mitochondrial Stress in Abdominal Aortic Aneurysm.” Clinical Science 133(13): 1421–1438. 10.1042/CS20190399. 31239294

[123] Qin, Y. , Y. Wang , O. Liu , L. Jia , W. Fang , J. Du , and Y. Wei . 2017. “Tauroursodeoxycholic Acid Attenuates Angiotensin II Induced Abdominal Aortic Aneurysm Formation in Apolipoprotein E‐Deficient Mice by Inhibiting Endoplasmic Reticulum Stress.” European Journal of Vascular and Endovascular Surgery 53(3): 337–345. 10.1016/j.ejvs.2016.10.026. 27889204

[124] Chen, B. , H. Zhou , X. Zhou , L. Yang , Y. Xiong , and L. Zhang . 2022. “Comprehensive Analysis of Endoplasmic Reticulum Stress in Intracranial Aneurysm.” Frontiers in Cellular Neuroscience 16: 865005. 10.3389/fncel.2022.865005. 35465608 PMC9022475

[125] Zhang, W. , M. Wang , K. Gao , X. Zhong , Y. Xie , L. Dai , W. Liu , et al. 2022. “Pharmacologic IRE1alpha Kinase Inhibition Alleviates Aortic Dissection by Decreasing Vascular Smooth Muscle Cells Apoptosis.” International Journal of Biological Sciences 18(3): 1053–1064. 10.7150/ijbs.63593. 35173538 PMC8771832

[126] Shi, F. , Z. Wang , Q. Wu , X. Zhong , M. Zhang , B. Li , W. Ren , S. Yuan , and Y. Chen . 2022. “Iron Deficiency Promotes Aortic Media Degeneration by Activating Endoplasmic Reticulum Stress‐Mediated IRE1 Signaling Pathway.” Pharmacological Research 183: 106366. 10.1016/j.phrs.2022.106366. 35882294

[127] McMonagle, M.P. 2020. “The Quest for Effective Pharmacological Suppression of Neointimal Hyperplasia.” Current Problems in Surgery 57(8): 100807. 10.1016/j.cpsurg.2020.100807. 32771085

[128] Braga, S. , J. Neves , J. Ferreira , C. Carrilho , J. Simões , and A. Mesquita . 2019. “Neointimal Hyperplasia.” Revista Portuguesa de Cirurgia Cardio‐Toracica e Vascular : orgao oficial da Sociedade Portuguesa de Cirurgia Cardio‐Toracica e Vascular 26: 213–217. 31734974

[129] Long, F. , D. Yang , J. Wang , Q. Wang , T. Ni , G. Wei , Y. Zhu , and X. Liu . 2021. “SMYD3‐PARP16 axis Accelerates Unfolded Protein Response and Mediates Neointima Formation.” Acta Pharmaceutica Sinica B 11(5): 1261–1273. 10.1016/j.apsb.2020.12.010. 34094832 PMC8148056

[130] Ishimura, S. , M. Furuhashi , T. Mita , T. Fuseya , Y. Watanabe , K. Hoshina , N. Kokubu , K. Inoue , H. Yoshida , and T. Miura . 2014. “Reduction of Endoplasmic Reticulum Stress Inhibits Neointima Formation after Vascular Injury.” Scientific Reports 4(1): 6943. 10.1038/srep06943. 25373918 PMC4221790

[131] Zeng, L. , Y. Li , J. Yang , G. Wang , A. Margariti , Q. Xiao , A. Zampetaki , et al. 2015. “XBP 1‐Deficiency Abrogates Neointimal Lesion of Injured Vessels via Cross Talk with the PDGF Signaling.” Arteriosclerosis, Thrombosis, and Vascular Biology 35(10): 2134–2144. 10.1161/ATVBAHA.115.305420. 26315405

[132] Ji, Y. , Y. Ge , X. Xu , S. Ye , Y. Fan , J. Zhang , L. Mei , et al. 2019. “Vildagliptin Reduces Stenosis of Injured Carotid Artery in Diabetic Mouse through Inhibiting Vascular Smooth Muscle Cell Proliferation via ER Stress/NF‐kappaB Pathway.” Frontiers in Pharmacology 10: 142. 10.3389/fphar.2019.00142. 30858802 PMC6397934

[133] Angbohang, A. , L. Huang , Y. Li , Y. Zhao , Y. Gong , Y. Fu , C. Mao , et al. 2021. “X‐Box Binding Protein 1‐mediated COL4A1s Secretion Regulates Communication between Vascular Smooth Muscle and Stem/progenitor Cells.” Journal of Biological Chemistry 296: 100541. 10.1016/j.jbc.2021.100541. 33722606 PMC8063738

[134] Pan, W. , W. Jie , and H. Huang . 2023. “Vascular Calcification: Molecular Mechanisms and Therapeutic Interventions.” MedComm 4(1). 10.1002/mco2.200. PMC9811665 36620697

[135] Li, Y. , Y. Li , Y. Li , Z. Yang , H. Geng , C. Liu , W. Hao , et al. 2021. “Inhibition of Endoplasmic Reticulum Stress Mediates the Ameliorative Effect of Apelin on Vascular Calcification.” Journal of Molecular and Cellular Cardiology 152: 17–28. 10.1016/j.yjmcc.2020.11.017. 33279504

[136] Liberman, M. , R. C. Johnson , D. E. Handy , J. Loscalzo , and J. A. Leopold . 2011. “Bone Morphogenetic Protein‐2 Activates NADPH Oxidase to Increase Endoplasmic Reticulum Stress and Human Coronary Artery Smooth Muscle Cell Calcification.” Biochemical and Biophysical Research Communications 413(3): 436–441. 10.1016/j.bbrc.2011.08.114. 21907184 PMC3197703

[137] Masuda, M. , T. C. Ting , M. Levi , S. J. Saunders , S. Miyazaki‐Anzai , and M. Miyazaki . 2012. “Activating Transcription Factor 4 Regulates Stearate‐Induced Vascular Calcification.” The Journal of Lipid Research 53(8): 1543–1552. 10.1194/jlr.M025981. 22628618 PMC3540843

[138] Cai, Z. , F. Li , W. Gong , W. Liu , Q. Duan , C. Chen , L. Ni , et al. 2013. “Endoplasmic Reticulum Stress Participates in Aortic Valve Calcification in Hypercholesterolemic Animals.” Arteriosclerosis, Thrombosis, and Vascular Biology 33(10): 2345–2354. 10.1161/ATVBAHA.112.300226. 23928865

[139] Duan, S. , X. Chen , Y. Liu , W. Guo , and W. Liu . 2022. “Endoplasmic Reticulum Stress Mediates Parathyroid Hormone‐Induced Apoptosis in Vascular Smooth Muscle Cells.” Renal Failure 44(1): 126–136. 10.1080/0886022X.2022.2027248. 35172689 PMC8856047

[140] Zhou, Y. , X. Wan , K. Seidel , M. Zhang , J. B. Goodman , F. Seta , N. Hamburg , and J. Han . 2021. “Aging and Hypercholesterolemia Differentially Affect the Unfolded Protein Response in the Vasculature of ApoE(‐/‐) Mice.” Journal of American Heart Association 10(18): e020441. 10.1161/JAHA.120.020441. PMC8649520 34533042

[141] Lee, H.‐Y. , H.‐K. Kim , T.‐H. Hoang , S. Yang , H.‐R. Kim , and H.‐J. Chae . 2020. “The Correlation of IRE1α Oxidation with Nox4 Activation in Aging‐Associated Vascular Dysfunction.” Redox Biology 37: 101727. 10.1016/j.redox.2020.101727. 33010578 PMC7530295

[142] Lee, H.‐Y. , H. M. A. Zeeshan , H.‐R. Kim , and H.‐J. Chae . 2017. “Nox4 Regulates the eNOS Uncoupling Process in Aging Endothelial Cells.” Free Radical Biology and Medicine 113: 26–35. 10.1016/j.freeradbiomed.2017.09.010. 28916474

[143] Matos, L. , A. M. Gouveia , and H. Almeida . 2015. “ER Stress Response in Human Cellular Models of Senescence.” J Gerontol A Biol Sci Med Sci 70(8): 924–935. 10.1093/gerona/glu129. 25149687

[144] Blazanin, N. , J. Son , A. B. Craig‐Lucas , C. L. John , K. J. Breech , M. A. Podolsky , and A. B. Glick . 2017. “ER Stress and Distinct Outputs of the IRE1alpha RNase Control Proliferation and Senescence in Response to Oncogenic Ras.” Proceedings of the National Academy of Sciences of the U S A 114(37): 9900–9905. 10.1073/pnas.1701757114. PMC5603998 28847931

[145] Banks, E. , G. Joshy , R. J. Korda , B. Stavreski , K. Soga , S. Egger , C. Day , N. E. Clarke , S. Lewington , and A. D. Lopez . 2019. “Tobacco Smoking and Risk of 36 Cardiovascular Disease Subtypes: Fatal and Non‐fatal Outcomes in a Large Prospective Australian Study.” BMC Medicine 17(1): 128. 10.1186/s12916-019-1351-4. 31266500 PMC6607519

[146] Song, M. , H. Peng , W. Guo , M. Luo , W. Duan , P. Chen , and Y. Zhou . 2019. “Cigarette Smoke Extract Promotes Human Lung Myofibroblast Differentiation by the Induction of Endoplasmic Reticulum Stress.” Respiration 98(4): 347–356. 10.1159/000502099. 31416082

[147] Zheng, C.‐M. , Y.‐H. Lee , I.‐J. Chiu , Y.‐J. Chiu , L.‐C. Sung , Y.‐H. Hsu , and H.‐W. Chiu . 2020. “Nicotine Causes Nephrotoxicity through the Induction of NLRP6 Inflammasome and Alpha7 Nicotinic Acetylcholine Receptor.” Toxics 8(4): 92. 10.3390/toxics8040092. 33114531 PMC7711477

[148] Gonzales, K. , V. Feng , P. Bikkina , M. A. Landicho , M. J. Haas , and A. D. Mooradian . 2021. “The Effect of Nicotine and Dextrose on Endoplasmic Reticulum Stress in Human Coronary Artery Endothelial Cells.” Toxicological Research 10(2): 284–291. 10.1093/toxres/tfab012. PMC8045581 33884179

[149] Adamopoulos, C. , E. Farmaki , E. Spilioti , H. Kiaris , C. Piperi , and A. G. Papavassiliou . 2014. “Advanced Glycation End‐Products Induce Endoplasmic Reticulum Stress in Human Aortic Endothelial Cells.” Clinical Chemistry and Laboratory Medicine 52(1): 151–160. 10.1515/cclm-2012-0826. 23454718

[150] Zhu, Y. , W. Ma , X. Han , Y. Wang , X. Wang , and N. Liu . 2018. “Advanced Glycation End Products Accelerate Calcification in VSMCs through HIF‐1alpha/PDK4 Activation and Suppress Glucose Metabolism.” Scientific Reports 8(1): 13730. 10.1038/s41598-018-31877-6. 30213959 PMC6137084

[151] Kosmopoulos, M. , D. Drekolias , P. D. Zavras , C. Piperi , and A. G. Papavassiliou . 2019. “Impact of Advanced Glycation End Products (AGEs) Signaling in Coronary Artery Disease.” Biochimica et Biophysica Acta, Molecular Basis of Disease 1865(3): 611–619. 10.1016/j.bbadis.2019.01.006. 30611860

[152] Guo, R. , W. Liu , B. Liu , B. Zhang , W. Li , and Y. Xu . 2015. “SIRT1 Suppresses Cardiomyocyte Apoptosis in Diabetic Cardiomyopathy: An Insight into Endoplasmic Reticulum Stress Response Mechanism.” International Journal of Cardiology 191: 36–45. 10.1016/j.ijcard.2015.04.245. 25965594

[153] Suzuki, R. , Y. Fujiwara , M. Saito , S. Arakawa , J. Shirakawa , M. Yamanaka , Y. Komohara , K. Marumo , and R. Nagai . 2020. “Intracellular Accumulation of Advanced Glycation End Products Induces Osteoblast Apoptosis via Endoplasmic Reticulum Stress.” Journal of Bone and Mineral Research 35(10): 1992–2003. 10.1002/jbmr.4053. 32427355

[154] Wu, L. , D. Wang , Y. Xiao , X. Zhou , L. Wang , B. Chen , Q. Li , X. Guo , and Q. Huang . 2014. “Endoplasmic Reticulum Stress Plays a Role in the Advanced Glycation End Product‐Induced Inflammatory Response in Endothelial Cells.” Life Sciences 110(1): 44–51. 10.1016/j.lfs.2014.06.020. 24997392

[155] Chen, J. , X. Hou , G. Wang , Q. Zhong , Y. Liu , H. Qiu , N. Yang , et al. 2016. “Terpene Glycoside Component from Moutan Cortex Ameliorates Diabetic Nephropathy by Regulating Endoplasmic Reticulum Stress‐Related Inflammatory Responses.” Journal of Ethnopharmacology 193: 433–444. 10.1016/j.jep.2016.09.043. 27664441

[156] Raymundo, D. P. , D. Doultsinos , X. Guillory , Antonio Carlesso , L. A. Eriksson , and E. Chevet . 2020. “Pharmacological Targeting of IRE1 in Cancer.” Trends Cancer 6(12): 1018–1030. 10.1016/j.trecan.2020.07.006. 32861679

[157] Cross, B. C. S. , P. J. Bond , P. G. Sadowski , B. K. Jha , J. Zak , J. M. Goodman , R. H. Silverman , et al. 2012. “The Molecular Basis for Selective Inhibition of Unconventional mRNA Splicing by an IRE1‐Binding Small Molecule.” Proceedings of the National Academy of Sciences of the U S A 109(15): E869–E878. 10.1073/pnas.1115623109. PMC3326519 22315414

[158] Sun, S. , Z. Ji , J. Fu , X. Wang , and L. S. Zhang . 2020. “Endosulfan Induces Endothelial Inflammation and Dysfunction via IRE1alpha/NF‐kappaB Signaling Pathway.” Environmental Science and Pollution Research International 27(21): 26163–26171. 10.1007/s11356-020-09023-5. 32361974

